# Exogenous incorporation of neugc-rich mucin augments n-glycolyl sialic acid content and promotes malignant phenotype in mouse tumor cell lines

**DOI:** 10.1186/1756-9966-28-146

**Published:** 2009-12-01

**Authors:** Mariano R Gabri, Laura L Otero, Daniel E Gomez, Daniel F Alonso

**Affiliations:** 1 Laboratory of Molecular Oncology, Quilmes National University, (Roque Saenz Peña 352), Bernal, (B1876BXD), Argentina

## Abstract

**Background:**

Carbohydrates embedded in the plasma membrane are one of the main actors involved in the communication of cells with the microenvironment. Neuraminic sialic acids are glycocalyx sugars that play important roles in the modulation of malignant cell behaviour. N-glycolylneuraminic acid (NeuGc) is synthesized by the cytidine monophospho-N-acetylneuraminic acid hydroxylase (CMAH), an enzyme expressed in all mammals except humans. In mice, this sugar is synthesized in several somatic tissues.

**Methods:**

We used the B16 melanoma and F3II mammary carcinoma mouse tumor cell lines. By CMAH directed RT-PCR and NeuGc detection with the specific anti-NeuGc-GM3 antibody 14F7 we evaluated enzyme and ganglioside expression in tumor cells, respectively. Expression of NeuGc-GM3 ganglioside was reached by *in vitro *incubation with NeuGc-rich bovine submaxillary mucin and evaluated by slot-blot and immunohistochemistry assays using the 14F7 antibody. Tumor cells treated with mucin or purified NeuGc were injected s.c. and i.v. in syngeneic mice to evaluate tumor and metastatic growth.

**Results:**

In the present work we demonstrated the absence of expression of CMAH enzyme in B16 melanoma and F3II mammary carcinoma cells. *In vitro *incubation of these NeuGc-negative cells with NeuGc-rich mucin increased the presence of NeuGc in cell membranes for at least 48-72 h, as a component of the GM3 ganglioside. Preincubation with NeuGc-rich mucin reduced tumor latency and increased the metastatic potential of tumor cells in syngeneic animals. Similar results were obtained when cells were incubated with purified NeuGc alone.

**Conclusion:**

Our results indicate that B16 and F3II mouse tumor cell lines do not express NeuGc in cell membranes but they are able to incorporate NeuGc from an exogenous source, contributing to the malignant phenotype of melanoma and mammary carcinoma cells.

## Background

The glycocalyx is composed of a broad variety of sugars that play a crucial role in the communication of cells with the microenvironment. Neuraminic sialic acids are 9-carbon sugars typically found in the glycocalyx that take part in the modulation of malignant cell behaviour [1,2]. They are usually found as a terminal component of different membrane glycoconjugates, such as glycoproteins or glycolipids. Major examples are mucins and gangliosides, both implicated in the modulation of cell behaviour [3,4]. The most common sialic acids in mammals are N-acetylneuraminic (NeuAc) and N-glycolylneuraminic (NeuGc) acids. The only structural difference between them consists of a single oxygen atom at the C-5 position of NeuGc catalyzed by the cytidine monophospho-N-acetylneuraminic acid hydroxylase (CMAH) [5]. While NeuGc is expressed in most somatic mouse cells, there is nearly no information regarding its expression in mouse cancer tissues [6]. Few reports suggest a null presence of this sugar in murine malignant cells.

Mucins are large molecular weight glycoproteins characterized by carbohydrate sugars attached via O-glycosidic linkages to serine or threonine, synthesized by a variety of secretory epithelial tissues as membrane-bound or secreted proteins. Characteristically, mucins present sialic acids as part of their sugar repertoire. In particular, the minor type of the bovine submaxillary mucin (BSM) presents a high concentration of NeuGc in its arborization [7].

It is well described that cells can process exogenous sialic acids from the extracellular environment and use them for their own glycoconjugates [8,9]. In this work we explored the impact of exogenous NeuGc incorporation by NeuGc-rich BSM or purified NeuGc *in vitro *incubation in the malignant behaviour of B16 melanoma and F3II mammary carcinoma mouse tumor cell lines.

## Methods

### Tumor cells

B16F0 and F3II cell lines were maintained in DMEM-F12 culture medium (Gibco BRL, Carlsbad, CA, USA) containing 10% heat-inactivated foetal bovine serum (FBS) (PAA, Pasching, Austria). Cells were subcultured twice a week using a trypsin-EDTA solution (Gibco BRL, Carlsbad, CA, USA). B16F0 is a C57BL/6 mouse melanoma cell line [10] while F3II is a mammary carcinoma cell line obtained from a clonal subpopulation of a spontaneous Balb/c mouse mammary tumor [11].

### RT-PCR

Expression of CMAH mRNA was evidenced by means of an RT-PCR assay, using total RNA from normal mouse liver or tumor cell lines as template. Total RNA was obtained using the RNAqueous Midi RNA kit (Ambion, Austin, TX, USA) following the manufacturer's instructions. RT reactions consisted of 5 μg total RNA, 10 mM dNTPs, 50 ng random hexamers (pd(N)_6_; GE Healthcare, Chalfont St. Giles, Buckinghamshire, England) as first strand primer, 0.1 M DTT, 40 U RNAseOUT (Invitrogen, Carlsbad, CA, USA) and 200 U Superscript III retrotranscriptase (Invitrogen, Carlsbad, CA, USA) in a 20 μl final volume. RT reactions were performed at 50°C during 1 h. The CMAH sequence was amplified by means of a PCR reaction comprised of 45 μl Supermix High Fidelity PCR mix (Invitrogen, Carlsbad, CA, USA), 10 pmol forward primer (5'-CGCCTTCCTGGTGTGA-3'), 10 pmol reverse primer (5'-GTTGGGTGGTGTTAGAGG-3'), and 1 μg cDNA obtained in the RT step. The amplification profile consisted of a single initial denaturation step (95°C, 5 min), followed by 35 cycles of 95°C, 30 seg; 53.7°C, 1 min and 72°C, 1.5 min; ending with a final extension step (72°C, 5 min). PCR reactions yielded the expected 1776 bp amplicon and also another two products with similar sizes. Accordingly with the publication of Koyama et al. [12] the expression of this enzyme results in splicing alternatives which can explain the alternative bands obtained in this work.

### Monoclonal antibodies

For immunohistochemistry or slot blot assays, the 14F7 monoclonal antibody was employed (gently provided by the Center of Molecular Immunology, Havana, Cuba). This murine IgG antibody has demonstrated a specific reactivity against NeuGc-GM3 ganglioside [13,14]. Additionally, Krengel et al. carried out a crystal structure analysis demonstrating that 14F7 specifically recognizes NeuGc-GM3, but not NeuAc-GM3 [15].

### Slot blot assay

Multiwell plates (9.6 cm^2^/well) were seeded with tumor cells (5 × 10^5 ^cells/well) in DMEM-F12 with 10% FBS. After 24 h, cells were incubated either with a fixed BSM concentration (250 μg/ml) during different time spans (24, 48 or 72 h) or with various BSM concentrations (250 or 125 μg/ml) for 24 h. The cell membrane fraction was obtained by an adaptation of the technique of Del Pozo et al. [16]. Briefly, cells were obtained on ice in a hypotonic buffer containing 10 mM Tris (pH 7.4), 5 mM MgCl_2_, 5 mM KCl, 1 mM DTT and 1× protease inhibitor cocktail (Invitrogen, Carlsbad, CA, USA). Cells were mechanically lysed with a glass homogenizer and centrifuged at 2,000 rpm. The supernatant was centrifuged at 15,000 rpm and the pellet was washed and resuspended in 100 μl of the hypotonic buffer. Total proteins were quantified by the Bradford assay (BioRad, Hercules, CA, USA). Identical masses of membrane fractions were seeded on a PVDF membrane (Hybond-P; GE Healthcare, Chalfont St. Giles, Buckinghamshire, England) previously activated with methanol and washed with TBS buffer with the aid of the BIO-DOT SP apparatus (Bio-Rad, Hercules, CA, USA). Once seeded, membranes were blocked with a 5% low-fat milk in TBS solution and washed with TBS. Incubation with the anti-NeuGc-GM3 antibody 14F7 (10 μg/ml) was performed at room temperature for 1 h. After washing them with TBS-T buffer, membranes were incubated with the biotinylated anti-mouse antibody (Vector Laboratories, Burlingame, CA, USA) and then incubated with a streptavidin linked to peroxidase solution (Vector Laboratories, Burlingame, CA, USA). Bands were detected by the ECL method (GE Heathcare, Chalfont St. Giles, Buckinghamshire, England) following the manufacturer's instructions. Membranes were analyzed with the ImageJ analysis software (National Institute of Health) and the intensity of each band was recorded and expressed as arbitrary units.

### Indirect immunoperoxidase staining

Tumor cells were cultured for 24 h in chamber-slides (Nalge-Nunc, Rochester, NY, USA) in serum-free DMEM-F12 medium containing 250 μg/ml of BSM (Sigma, St. Louis, MO, USA), and later formalin-fixed. Subsequently, monolayers were stained by the Vectastain kit (Vector Laboratories, Burlingame, CA, USA) according to the manufacturer's instructions. 14F7 mAb was used as primary antibody at a concentration of 10 μg/ml. Cells were counterstained with hematoxylin.

### Adhesion assay

B16 or F3II cells were seeded (40,000 cells/well) in 96-well plates in D-MEM supplemented with 2 or 5% FBS, in the presence or absence of 50-100 μg/ml of purified NeuGc (Sigma, St. Louis, MO, USA). Cells were incubated at 37°C in a CO_2 _incubator for 60 min. After incubation, cells were washed twice with 1× PBS buffer and fixed with methanol (100 μl/well). After a 10-min incubation, cells were stained with a 0.1% crystal violet solution (100 μl/well) for 10 min. After washing thoroughly with distilled water, 60 μl/well of a 10% methanol-5% acetic acid solution were added and the plate was shook for a few minutes. Absorbance at 595 nm was measured.

### Proliferation assay

B16 or F3II cells were seeded (2,500 cells/well) in 96-well plates in D-MEM supplemented with 1, 5 or 10% FBS, in the presence or absence of 50-100 μg/ml of purified NeuGc. Plates were incubated at 37°C in a CO_2 _incubator for 72 h. After incubation, cells were treated with MTT (0.5 mg/ml 3-(4,5-dimethylthiazol-2-yl)-2,5-diphenyltetrazolium bromide in PBS buffer; Sigma, St. Louis, MO, USA). After a 3-h incubation, the supernatant was discarded, cells were resuspended in DMSO and absorbance was measured at 570 nm.

### *In vivo *inoculation of BSM or NeuGc-preincubated cells into syngeneic mice

Tumor cell suspensions were preincubated with 500 μg/ml of BSM or 100 μg/ml of NeuGc in culture medium for 1 h and then extensively washed and resuspended. Control cells were incubated in the same medium without the addition of BSM or NeuGc. Inbred C57BL/6 and Balb/c mice were inoculated intravenously with 1 × 10^5 ^B16 and F3II cells, respectively. After 22 days, lungs were collected, fixed in Bouin's solution, and metastasic foci were counted under a dissecting microscope. In another set of experiments, mice were injected subcutaneously with B16 tumor cells preincubated or not with BSM. The time of appearance of local tumors was monitored by palpation and further confirmed by histopathology. Tumor size was measured with a caliper twice a week and tumor diameter was calculated as the square root of width × length. Animals were sacrificed 60 days after tumor inoculation or when they became moribund.

## Results

**Figure 1:**
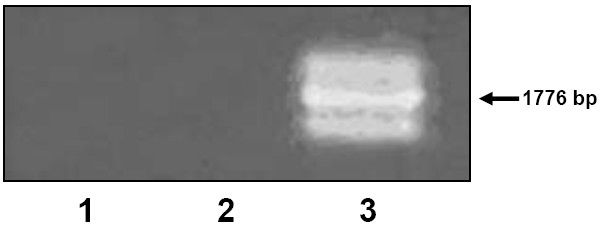
**Expression of the CMAH mRNA evidenced by RT-PCR**. Lane **1**, total RNA from the B16 mouse melanoma cell line; lane **2**, total RNA from the F3II mouse mammary carcinoma cell line; lane **3**, total RNA from normal mouse liver.

We first checked the expression of CMAH in B16 melanoma and F3II mammary carcinoma cells. To assess the presence of CMAH mRNA, an RT-PCR assay using high affinity primers was performed. As expected, normal liver was positive for CMAH expression, but neither B16 nor F3II cells expressed the gene. When performed on total RNA from normal liver, the RT-PCR assay yielded 3 distinct products (Fig. 1). After sequencing, all 3 shared a very high homology with the CMAH gene sequence. The intermediately-sized amplicon shared a 99% identity with the CMAH sequence while the other two proved to be alternatively spliced variants, as reported by Koyama *et al *[12].

**Figure 2:**
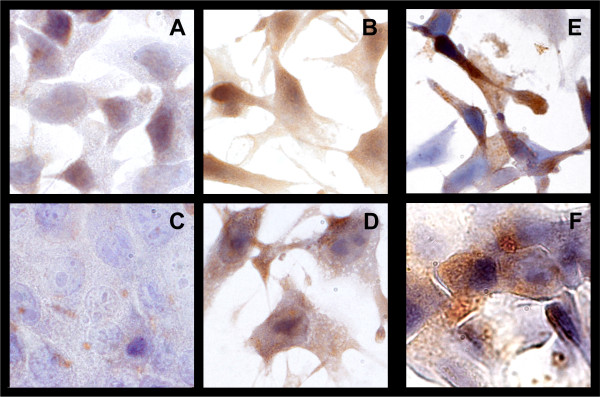
**Indirect immunoperoxidase staining of the NeuGc-GM3 ganglioside with 10 μg/ml of 14F7 monoclonal antibody on formalin-fixed B16 (A, B and E) and F3II (C, D and F) monolayers, cultured in the presence (B and D) or absence (A and C) of 10% FBS or incubated with 250 μg/ml mucin in FBS-free medium for 24 h (E and F)**. Original magnification 1000×.

We then examined the expression of NeuGc in tumor cells by immunohistochemical staining, using the 14F7 antibody reactive against NeuGc-GM3. No expression was detected under serum-free *in vitro *culture conditions. On the contrary, in the presence of FBS both B16 and F3II cells became clearly positive (Fig. 2A-D), suggesting that NeuGc can be incorporated from the bovine source.

**Figure 3:**
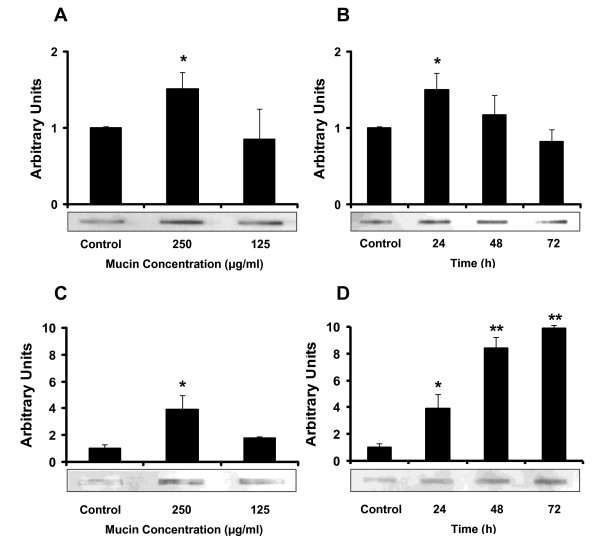
**Detection of NeuGc-GM3 in cell membrane fraction by slot blot assay in B16 (A and B) and F3II (C and D) cells**. **A **and **C**, tumor cells were preincubated with different concentrations of NeuGc-rich BSM and processed 24 h later. **B **and **D**, tumor cells were preincubated with 250 μg/ml of NeuGc-rich BSM and further processed 24, 48 or 72 h after preincubation. In all cases, densitometric analysis was normalized to the respective control. Means ± SEM of at least 3 determinations are shown. *p < 0.05, **p < 0.01 (ANOVA contrasted with Dunnet test).

In order to increase NeuGc density in the cell membrane, we incubated B16 and F3II cells *in vitro *with the minor type of BSM, a mucin fraction with high NeuGc content [7]. As expected, *in vitro *cell incubation with NeuGc-rich BSM changed the ganglioside expression profile of B16 and F3II cells resulting in a significant increase of NeuGc-GM3 presence in cell membranes, as revealed by slot blot analysis (Fig. 3A and 3C). The expression was maintained in mouse tumor cells for at least 48-72 h (Fig. 3B and 3D). The same result was observed by immunohistochemical staining with 14F7 antibody on *in vitro *monolayer cultured cells (Fig. 2E and 2F).

**Figure 4:**
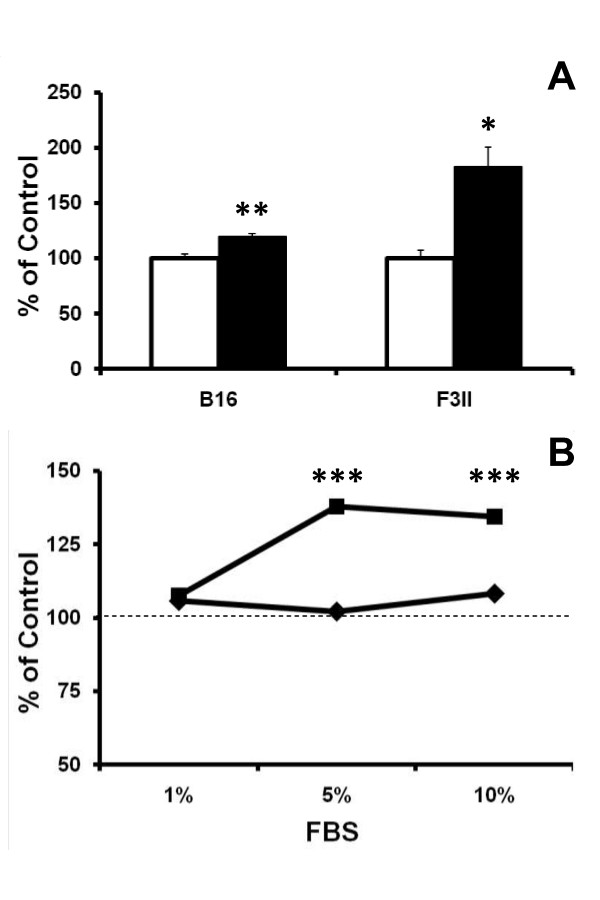
**A, adhesion assay**. B16 or F3II cells were incubated for 1 h in medium with 2% FBS, either with (filled bars) or without (empty bars) 50 μg/ml of purified NeuGc. Data represent mean ± SEM (n = 6). *p < 0.05, **p < 0.01 (t test). **B**, proliferation assay. B16 (black square) or F3II (black diamond) cells were grown for 72 h in medium supplemented with 1, 5 or 10% FBS, either with or without 100 μg/ml of purified NeuGc. Dashed line refers to proliferation in control monolayers without addition of NeuGc. Data represent mean of 6 determinations; in all cases SEM was less than 5%. ***p < 0.001 versus the respective control (ANOVA contrasted with Tukey-Kramer multiple comparisons test).

Interestingly, incubation of tumor cells with purified NeuGc modulates the *in vitro *behaviour. Tumor cell adhesion showed a significant increase in both cell lines (Fig 4A); while NeuGc addition impacted differently on proliferation, significantly increasing growth in B16 but not in F3II cells (Fig 4B).

Finally, we evaluated tumorigenicity and lung colonization of BSM-preincubated tumor cells in syngeneic mice. In both mouse models preincubation with NeuGc-rich BSM significantly enhanced the metastatic ability of tumor cells, approximately doubling the number of lung nodules after intravenous cell injection (Table 1). Similar results were obtained after preincubation with purified NeuGc. B16 NeuGc-treated cells showed a 65% increase in lung nodules (Control: 14.5 ± 4.8, NeuGc: 22.3 ± 3.8; p = 0.14, Mann-Whitney U test), while for F3II NeuGc-treated cells the number of lung nodules resulted in a 112% increase (Control: 7.3 ± 1.8, NeuGc: 15.5 ± 2.2; p < 0.05, Mann-Whitney U test). Although all animals challenged in the flank developed subcutaneous tumors, we observed a rapid tumor take with BSM-preincubated B16 cells. Significant differences were obtained for tumor latency and size of melanoma tumors. However, preincubation with BSM did not significantly modify tumor growth rate (Table 2).

**Table 1:** Experimental lung metastasis in syngeneic mice after intravenous injection of B16 melanoma or F3II mammary carcinoma cells, preincubated or not with NeuGc-rich BSM.

Treatment	Lung metastasis^A^ (nodules per animal)	
	
	B16 cells	F3II cells
Control	6.4 ± 2.2	6.2 ± 2.1
BSM-preincubated	11.6 ± 1.5*	13.3 ± 3.1*

^A^Lung nodules were counted 22 days after intravenous injection of B16 or F3II cells (1 × 10^5 ^cells/mouse). Values represent mean ± SEM of at least 10 mice.

*p < 0.05 versus the respective control (Mann-Whitney U test).

**Table 2:** Latency and size of melanoma tumors after inoculation of B16 cells, preincubated or not with NeuGc-rich BSM.

Treatment	Tumor latency^A^ (days)	Tumor Diameter^A^ (mm)	Tumor Growth Rate^A^ (mm/day)
Control	12.8 ± 1.6	2.2 ± 0.9	0.15 ± 0.03
BSM-preincubated	8.4 ± 0.6**	7.1 ± 1.8*	0.18 ± 0.05

^A^Tumor latency represents the time between the subcutaneous injection of a small burden of B16 cells (5 × 10^3 ^cells/mouse) and the appearance of detectable tumors.

Tumor diameter was recorded at day 35 after tumor cell inoculation. Values represent mean ± SEM of at least 8 mice.

**p < 0.01 and *p < 0.05 (t test).

## Discussion

NeuGc and NeuAc are two of the main sialic acids in mammals, being the presence of the oxygen atom in the C-5 position the single difference between them. This seemingly minor difference is crucial in many aspects of cellular behaviour and is produced solely by the CMAH enzyme [5,17]. This enzyme is present in animals from the deuterostome lineage [18], which includes all higher mammals. The expression of this particular enzyme is the reason for NeuGc presence in most murine normal tissues [19,20]. In humans, an exon deletion/frameshift mutation in the CMAH gene renders the major pathway for NeuGc production non functional [21].

Sialic acids have been associated with intrinsic receptors that function as ligands for specific leucocyte receptors [22,23] or as extrinsic receptors themselves for certain pathogens [24,25]. The presence of the distinctive oxygen atom in NeuGc is determinant in the relationship of the cell with specific molecules or viruses [26,27]. As an example, mouse CD22 (Siglec-2), a regulator of B-cell signalling, homeostasis and survival presents high affinity for NeuGc whereas its affinity for NeuAc is low [23].

Exploring the expression of NeuGc in murine cell lines, we have found that B16 and F3II cell lines do not express the CMAH gene and therefore under-express NeuGc in their cell membranes. Considering that most normal mouse somatic cells are positive for the expression of this gene, it is an interesting fact that malignant cells lack such expression. In cancer, sialic acids are over-expressed as part of gangliosides in several malignancies and their involvement in the malignant cell behaviour has been previously reported [28-30]. The lack of expression of NeuGc in mouse tumor cells suggests that the silencing of the CMAH gene is an important step in the cell transformation process in this specie. Ecsedy et al. [31] reported that two brain tumor cell lines express this enzyme under *in vivo *conditions but not *in vitro*. However, authors could not discard potential contamination of *in vivo *samples with RNA from lymphoid cells, which have demonstrated to be positive for CMAH expression [32].

In human cancer, the situation is dramatically different. Interestingly, considering the null expression of NeuGc in human somatic cells, the expression of NeuGc-GM3 in some human tumors was undoubtedly found [33-35]. Yin et al. reported notable results supporting the idea that tumor hypoxia could be one of the factors responsible for the presence of the non-human sialic acids, such as NeuGc, in human tumors [36].

It is known that cells are able to take in and process exogenous sialic acids for their own glycoconjugates [8,9]. In our work, the cell lines tested were able to express NeuGc-GM3 when cultured in the presence of serum, suggesting an active incorporation of the sugar residue from the culture medium. Taking this fact into account, we incubated tumor cells with a NeuGc-rich fraction of BSM [7], looking for an increase in NeuGc presence in the cell membrane. Our results show that this strategy renders a transient increase of NeuGc-GM3 presence in the cell membrane, indicating endocytosis of BSM components, with consequent processing and utilization of NeuGc. In control slots, a slight staining with 14F7 antibody was observed. As it was demonstrated, this recognition could be due to the previous acquisition of NeuGc from bovine serum present in the growth medium during standard cell culture conditions. Numerous experiments have shown that mucin expression in tumor cells can enhance malignant behaviour [37,38]. However, there are no reports showing that these molecules are able to be taken in and processed by cells. Our results support the idea that cells are able to process the NeuGc-rich BSM, incorporating some of their components in the carbohydrate sugar chains of the plasma membrane. Expression of NeuGc-GM3 on cell membrane as a consequence of preincubation with NeuGc-rich culture medium, was demonstrated also by immunohistochemistry. Results support that NeuGc present in culture medium can be incorporated and expressed on the cells either coming from bovine serum or from mucin.

The altered sugar expression pattern obtained after incubation with NeuGc-rich BSM or purified NeuGc resulted in promotion of the malignant phenotype. Preincubation with BSM or NeuGc increased the metastatic ability of both B16 melanoma and F3II carcinoma cells, and a reduced melanoma tumor latency by BSM preincubation was also observed. As it was shown, the presence of NeuGc in the plasma membrane is maintained *in vitro *for no more than two or three days. It is expected that an equal decline in the expression takes place *in vivo*. To explore the impact of NeuGc presence on tumor growth, we are currently working on the development of CMAH transfected cell lines in order to obtain a stable NeuGc expression tumor model. Preliminary results (not shown) suggested that transfected tumor cells have an increased *in vitro *adhesion and proliferation in a similar manner as mucin or NeuGc-treated cells. Since NeuGc-GM3 is a postulated tumor antigen in human cancers [39], development of NeuGc-positive murine tumor cells allows the possibility to evaluate cancer vaccines in animal models [40].

Considering the results obtained we hypothesize that NeuGc presence in the cell membrane is actively involved in the early phases of tumor formation and takes part in tumor nesting at distant sites.

## Competing interests

The authors declare that they have no competing interests.

## Authors' contributions

MRG participated in the design and coordination of the study, LLA carried out most of the experiments, DEG and DFA conceived the study. All authors read and approved the final manuscript.
